# Association between protein intake, diet quality, and obesity in Australian adults: a comparison of measurement units

**DOI:** 10.1017/jns.2024.56

**Published:** 2024-09-20

**Authors:** Hesti Retno Budi Arini, Rebecca M. Leech, Sze-Yen Tan, Sarah A. McNaughton

**Affiliations:** 1 Institute for Physical Activity and Nutrition (IPAN), School of Exercise and Nutrition Sciences, Deakin University, Geelong, Australia; 2 School of Exercise and Nutrition Sciences, Deakin University, Geelong, Australia; 3 Health and Well-Being Centre for Research Innovation, School of Human Movement and Nutrition Sciences, University of Queensland, St Lucia, Australia

**Keywords:** Dietary proteins, Diet quality, Obesity, Unit

## Abstract

Previous investigations on protein associations with diet quality and obesity still have inconclusive findings, possibly due to how protein intake was expressed. This study aimed to compare how different ways of expressing total protein intake may influence its relationships with diet quality and obesity. Usual protein intake was estimated from the 2011–12 Australian National Nutrition and Physical Activity Survey (*n* = 7637 adults, ≥19 years), expressed in grams (g/d), percent energy (%EI), and grams per actual kilogram body weight (g/kgBW/d). Diet quality was assessed using the 2013 Dietary Guidelines Index, and obesity measures included Body Mass Index (BMI) and waist circumference (WC). Sex-stratified multiple linear and logistic regressions were performed and adjusted for potential confounders. Total protein (g/d) was directly associated with diet quality (males, β = 0.15 (95% CI 0.12, 0.19); females, β = 0.25 (0.22, 0.29)), and this association was consistent across units. Protein intake (g/d) was directly associated with BMI (males, β = 0.07% (0.04%, 0.11%); females, β = 0.09% (0.04%, 0.15%)), and WC (males, β = 0.04 (0.01, 0.06); females, β = 0.05 (0.00, 0.09)). While in males, protein as %EI was associated with higher WC, no association was found in females. Adults with higher protein intake (g/d) had higher odds of overweight/obesity (males, OR = 1.01 (1.00, 1.01); females, OR = 1.01 (1.00, 1.01)), and central overweight/obesity (females, OR = 1.01 (1.00, 1.01)), but no significant association with females odds of overweight/obesity when protein was expressed in %EI. In conclusion, protein intake was positively associated with diet quality and obesity, yet these associations were stronger for women. The effect sizes also varied by measurement unit due to the different scales of those units.

## Introduction

The global burden of cardiometabolic diseases is increasing and is mainly attributed to modifiable risk factors.^(1,2)^ The global cases of cardiovascular diseases (CVDs) almost doubled from 271 million to 523 million in the 1990–2019 period,^(1)^ while diabetes cases were predicted to increase from 476 million in 2017 to 570.9 million in 2025.^(2)^ About one-third of Australians’ disability-adjusted life years were attributed to modifiable risk factors, such as dietary risk factors and high body mass,^(3)^ and the total CVD burden was attributed to several cardiometabolic risk factors including high blood pressure (36%), dietary risk (31%), and overweight/obesity (22%).^(4)^

Preventing obesity and related diseases requires successful weight management, in which protein plays a significant role. Protein has the most satiating effect compared to carbohydrates and fat, following the large increase of appetite-suppressing hormones (e.g. glucagon-like peptide-1) and the marked decrease of appetite-stimulating hormones (e.g. ghrelin).^(5)^ In addition to its appetite-regulating effects, larger weight loss following high-protein diets is also attributed to the larger thermic effect, compared to other macronutrients.^(5,6)^ High-protein diets can also delay obesity by preventing hyperphagia, particularly after high-fat feeding.^(7)^

Despite inconclusive associations between diet quality indices and obesity,^(8)^ a growing number of studies reported a consistent association between protein intake and diet quality. Higher diet quality as assessed in observational studies using a variety of scores, such as the Healthy Eating Index (HEI), the Dietary Guidelines Index (DGI), and the Mediterranean-Dietary Approaches to Stop Hypertension Diet Intervention for Neurological Delay Index (MIND), were observed among American, Australian, and French adults consuming more protein.^(9–15)^ Protein intake in those studies was expressed in different units, such as gram/day (g/d), per cent of energy intake (%EI), and g/kg body weight (BW)/d, but no single study compared the influence of ways of expressing protein intake on the association.

Each way of expressing protein intake, that is the protein units, may result in different interpretations of adequacy. For example, the current protein intake recommendation for adults is 0.83 g/kgBW/d, with the acceptable BW being based on either actual BW or median weight-for-height.^(16)^ When those references are applied to individuals with obesity consuming adequate grams of protein, they will still be considered to have inadequate protein intake as the ratio between their absolute intake and BW is still lower than the reference value.^(17)^ In fact, this dissimilarity was likely because of their higher actual BW or energy intake rather than absolute protein inadequacy.^(17)^

The association between protein intake with BMI and other cardiometabolic outcomes also varied across different units. The unit of g/d, %EI, and g/ideal BW/d suggested direct associations, with some inconsistencies across models, but the g/actual kgBW/d unit consistently produced inverse associations, which were considered spurious.^(18)^ Hence, given the lack of consensus on which units should be used in examining protein association with diet quality and obesity, this study aimed to compare how different total protein units are associated with diet quality and obesity.

## Methods

### Sample and study design

This study was a secondary data analysis from the Australian National Nutrition and Physical Activity Survey (NNPAS) 2011–12, which was conducted across eight states and territories by the Australian Bureau of Statistics (ABS).^(19)^ The survey design was a stratified multistage area of private dwellings with a probability sampling design.^(19)^ The NNPAS included 12153 participants, but this study only focused on adults aged ≥19 years (*n* = 9341). Participants who identified themselves as pregnant and lactating women were excluded accounting for their possibility to consume restricted or unusual diets and potential impact on diet quality and weight. Adult participants with no data on anthropometric or dietary measurement were also excluded, which resulted in 7637 adults being analysed in this study.

### Ethics statement

This study was conducted according to the guidelines laid down in the Declaration of Helsinki. The ethics approval for the ABS in conducting surveys, including the interview component of the NNPAS, was provided through the Census and Statistics Act 1905.^(19)^ Informed consent was sought from all individual participants through the completion of a consent form.^(19)^ All secondary data analyses in this study were conducted using deidentified data, and exemption from ethics review has been approved by the Deakin University Human Research Ethics Committee (DUHREC no. 2023-135).

### Dietary assessment

The dietary data of NNPAS was collected through Computer Assisted Personal Interview (CAPI) for the first 24-h recalls by trained interviewers.^(19)^ At least 8 d after CAPI, approximately 65% of overall adult survey respondents participated in the second 24-h recalls through Computer-Assisted Telephone Interview (CATI).^(19)^ The 24-h recall adopted the USDA Automated Multiple 5-Pass Method, which divided the interview into five phases.^(19)^ For each 24-h recall, participants were requested to report foods and beverages, eating occasions, amount, and time of consumption.^(19)^ Further details were also probed, including brand names and preparation methods.^(19)^ Each food and beverage was later coded, followed by the calculations of energy and nutrient intakes referring to the AUSNUT13 food nutrient database that comprises fifty-three nutrients.^(20)^

### Protein and energy intake

Total protein (g) and energy intake (kJ) information was obtained from the dietary intake data of NNPAS, which was estimated from all food and beverage items. Dietary data from both 1- and 2-d 24-h recall was modelled using the Multiple Source Method^(21)^ to estimate usual dietary intake. Total energy, protein, fat, and carbohydrates were modelled separately, with a number of recall days, age, sex, and age–sex interaction terms included in the models. Total protein intake in this study was reported in three measurement units, namely, g/d, g/kgBW/d, and %EI, considering their wide use to evaluate protein intake adequacy and assess protein contribution to total energy intake.

Australian Nutrient Reference Value used the age/gender-standardised BWs from the 1995 National Nutrition Survey,^(22)^ which was similar to the standardised BWs of the US Dietary Reference Intake published prior to 2002.^(23)^ Therefore, protein and other nutrient intake recommendations for Australian adults were based on standard BWs for the 19–30 years age group.^(22)^ However, given that adult BW in most western populations is likely to increase because of increasing body fat,^(22)^ g/kgBW/d in this study was calculated based on participants’ actual BWs.

### Diet quality

Diet quality was assessed using the DGI to measure adults’ compliance with the 2013 Australian Dietary Guidelines.^(24–26)^ The DGI consists of seven recommended dietary components (i.e. variety of foods, fruits, vegetables, cereals, meat and other high-protein foods, dairy products, and water) and six discouraged components (i.e. saturated fat, unsaturated fat, added salt, added sugar, discretionary foods, and alcohol).^(25,26)^ Each item is scored between 0 and 10, resulting in 0–130 DGI scores with a higher score suggesting better diet quality.^(26)^

DGI score in this study was calculated using the disaggregated foods from ABS data, including fruit, vegetable, and protein food groups.^(19)^ All DGI calculations only included non-discretionary foods, except for the discretionary foods component. Food variety was calculated using grams of consumed fruits, vegetables, whole grains, low-fat dairy, and lean meats and alternatives, as described elsewhere.^(27)^ Number of daily servings was directly used to calculate DGI scores of fruits and vegetables, while grains and cereals score was calculated from daily servings of total cereals and whole-grain and refined-grain breads. Meats and alternatives scores were based on red meats, poultry, fish, eggs, nuts, tofu, legumes, and beans consumption, while dairy intake comprised milk, yoghurts, cheese, and alternatives. Total beverages included water, milk and soy beverages, smoothies, juices, low-calorie cordials and soft drinks, tea, and coffee.

The number of discretionary food servings was obtained by dividing energy intake from discretionary foods by 600 kJ.^(28)^ Saturated fat intake score was estimated against lean red meats and poultry (<10% fat), and low-fat milk, while unsaturated fat intake included margarine, seeds, and nuts.^(27)^ Added salt intake score was based on NNPAS questions on whether salt is added during cooking and meals.^(25)^ To obtain the number of servings per day, grams of added sugar and alcohol intake were divided by five and ten, respectively.^(20,28)^ The detailed construction of each DGI score component was provided in Supplementary Material 1.

### Anthropometric measurements

BW, height, and waist circumference (WC) measurements were conducted by trained ABS staff during the interview.^(19)^ BW was measured using digital scales (max. 150 kg), and a stadiometer (max. 210 cm) was used for height measurement.^(19)^ A metal tape measure (max. 200 cm) was used to measure WC, and 10% of participants were randomly selected for additional measurement to validate the collected height and WC measurement data.^(19)^ BMI scores were calculated by dividing weight (kg) by squared height (m)^2^.^(19)^ The overweight/obesity status was determined using BMI and WC as binary variables. Individuals with BMI ≥ 25 kg/m^2^ were categorised as having overweight/obesity. Drawing on ABS categories of WC, female individuals with WC ≥ 80 cm or male individuals with WC ≥ 94 cm were classified as having central overweight/obesity.^(19,29)^

### Covariates

Age, country of birth, Socio-economic Indexes for Areas (SEIFA), and physical activity level (PAL) were used as covariates, based on the previous literature.^(15,30–33)^ Age data were reported in years, while country of birth was categorised as (a) Australia; (b) Mainly English-speaking countries; and (c) Other.^(19)^ SEIFA ranked Australia’s areas according to relative socioeconomic advantage and disadvantage, where the lower quintile indicated greater disadvantage.^(34)^ Participants’ PAL was categorised as meeting and not meeting physical activity guidelines of 150 min and 5 sessions/week.^(19)^ This cut-off was based on Australia’s Physical Activity and Sedentary Behaviour Guidelines, which recommend adults to regularly perform moderate-to-vigorous-intensity physical activity and strength training.^(35)^

### Energy misreporting

Energy misreporting was estimated in this study due to previous findings on energy and protein underreporting in self-reported dietary intake.^(36)^ Energy misreporting was examined using the ratio between reported energy intake (rEI) and predicted total energy expenditure (pTEE; rEI:pTEE), as has been done in previous studies.^(37,38)^ pTEE was calculated using the validated equations of the Institute of Medicine, which considered sex, age, height, BW, and physical activity.^(39)^ A low-active PAL was assumed (1.4 ≤ PAL < 1.6) because the NNPAS did not measure overall physical activity.^(37,38)^ Participants were categorised as plausible reporters, underreporters, or overreporters of energy intake based on the published calculations for the ±1 SD cutoff for rEI:pTEE, incorporating the coefficient of variation (CV) of rEI, pTEE, and the technical error of measuring total energy expenditure (mTEE).^(37,38)^ Among NNPAS adult participants, the CV_rEI_ for those having 1- and 2-d 24-h recall was 43.2% and 34.5%, respectively. The CV_pTEE_ was 17.6% for NNPAS adult participants with 1-d 24-h recall and 17.7% for those with 2 recall days. The CV_mTEE_ was 8.2%, as estimated by previous research using the doubly labelled water method.^(40)^ This equation resulted in ±1 SD cutoff of 47% and 31% for individuals having 1- and 2-d 24-h recall, respectively.

### Statistical analysis

Statistical analyses were performed using Stata v.17, with P < 0.05 considered statistically significant for all analyses. All statistical analyses in this study used the benchmarked survey weight to produce estimates for the population (i.e. replicate weights and person-level weights). Descriptive statistics comprising proportions and means with standard deviation were used to examine protein intake data, with differences across protein intake tertiles expressed in g/d were tested using one-way ANOVA and Chi-square test. All statistical analyses were stratified by sex to account for differences in protein requirements between men and women.^(24)^

The association between protein intake, expressed using the three different measurement units (g, % EI, g/kBW/d), and diet quality was assessed using separate multiple linear regression models. Model 1 was adjusted for age (continuous), country of birth (categorical), SEIFA (categorical), and PAL (categorical), while Model 2 was additionally adjusted for usual non-protein energy intake (continuous). The influence of energy misreporting on the association was examined in Model 3 by additionally adjusting for energy misreporting status (categorical).

Several tests were performed to test if linear regression models satisfy the assumptions of ordinary least squares linear regression. Added-value plots were used to check linear relationships between variables, and models were tested for multicollinearity using variance inflation factor. At this stage, usual fat and carbohydrate intakes were excluded from all models due to the multicollinearity issue (VIF > 5), and BMI outcome was natural-log transformed before stratification to improve the normality of residuals. Jackknife standard errors were estimated in the models to address heteroscedasticity as shown by both graphical (i.e. rvfplot command) and non-graphical (i.e. hettest command) methods.^(41)^

To investigate protein intake associations with obesity, multiple linear regression was conducted between total protein intake and continuous BMI and WC, and multiple logistic regression was conducted between total protein intake and binary variables of overweight/obesity and central overweight/obesity. Despite the wide use of g/kgBW/d unit in expressing protein intake recommendations,^(16)^ both linear and logistic regressions were only performed using the units of g/d and %EI due to the potentially spurious associations between protein intake expressed in g/kgBW/d, cardiometabolic health, and body composition.^(18,42)^ The first linear regression model for BMI and WC was adjusted for age (continuous), country of birth (categorical), SEIFA (categorical), and PAL (categorical). Model 2 was additionally adjusted for usual non-protein energy intake (kJ, continuous) to address the conditional dependency between protein and other macronutrients in causing obesity and to minimise the composite variable bias due to protein contribution to total energy intake.^(43,44)^ For these reasons, a sensitive analysis adjusting for the usual total energy intake was also conducted and provided in Supplementary Material 2. Model 3 was the same as Model 2 with further adjustment for energy misreporting status (categorical). Using the same adjustments, three multiple logistic regression models were performed with binary variables of BMI and WC as the outcomes.

## Results

A total of 7637 individuals were included in this study as summarised in Table 1 (male, *n* = 3684; female, *n* = 3953). There were significant differences in the characteristics of adult Australian males across protein intake tertiles. However, the country of birth and obesity measures of Australian females were not significantly different across tertiles. In both sexes, higher protein intake was associated with higher usual total energy intake, while most energy misreporters were in the lowest tertile of protein intake. Both males and females in the lowest tertile of protein intake had the lowest DGI score, and the detailed component score of DGI was provided in Supplementary Material 3.

**Table 1.** Descriptive characteristics of adults (N = 7637) by tertiles of total protein intake (g/d)^a^

	Male (3684 (48.2))				Female (3953 (51.8))			
	T1	T2	T3	**Pvalue**	T1	T2	T3	P value
**Demographic characteristics**								
Age (year, mean (SD))	50.5 (17.7)	48.7 (16.9)	44.5 (15.8)	<0.001	50.0 (18.4)	50.0 (17.2)	48.3 (16.5)	0.02
Country of birth (*n* (%))				<0.001				0.20
Australia	807 (65.7)	893 (72.7)	856 (69.7)		914 (69.3)	960 (72.8)	963 (73.1)	
Mainly English-speaking countries	174 (14.2)	166 (13.5)	151 (12.3)		167 (12.7)	154 (11.7)	146 (11.1)	
Other	247 (20.1)	169 (13.8)	221 (18.0)		237 (18.0)	204 (15.5)	208 (15.8)	
SEIFA (*n* (%))				0.004				<0.001
Q1 (most disadvantaged)	252 (20.5)	206 (16.8)	198 (16.1)		292 (22.2)	257 (19.5)	219 (16.6)	
Q2	272 (22.1)	240 (19.5)	242 (19.7)		291 (22.1)	252 (19.1)	268 (20.3)	
Q3	233 (19.0)	249 (20.3)	257 (20.9)		253 (19.2)	246 (18.7)	294 (22.3)	
Q4	220 (17.9)	214 (17.4)	249 (20.3)		218 (16.5)	244 (18.5)	207 (15.7)	
Q5 (least disadvantaged)	251 (20.4)	319 (26.0)	282 (23.0)		264 (20.0)	319 (24.2)	329 (25.0)	
Physical activity (*n* (%))				0.006				0.002
Met physical activity guidelines	503 (41.0)	554 (45.1)	580 (47.2)		506 (38.4)	556 (42.2)	596 (45.3)	
Did not meet physical activity guidelines	725 (59.0)	674 (54.9)	648 (52.8)		812 (61.6)	762 (57.8)	721 (54.7)	
BMI (kg/m^2^, mean (SD))	28.1 (5.0)	27.9 (4.8)	27.6 (4.5)	0.01	27.5 (6.3)	27.1 (6.0)	27.3 (6.2)	0.28
Obesity status (*n* (%))				0.02				0.46
Overweight/obese^b^	909 (74.0)	878 (71.5)	848 (69.1)		785 (59.6)	755 (57.3)	761 (57.8)	
Not overweight/obese	319 (26.0)	350 (28.5)	380 (30.9)		533 (40.4)	563 (42.7)	556 (42.2)	
WC (cm, mean (SD))	99.3 (13.2)	98.5 (13.0)	97.2 (12.5)	<0.001	89.1 (14.9)	88.2 (14.7)	88.9 (14.6)	0.29
Central obesity status (*n* (%))				<0.001				0.15
Centrally overweight/obese^c^	807 (65.7)	781 (63.6)	685 (55.8)		925 (70.2)	900 (68.3)	945 (71.8)	
Not centrally overweight/obese	421 (34.3)	447 (36.4)	543 (44.2)		393 (29.8)	418 (31.7)	372 (28.2)	
Dietary intake								
Usual energy intake (kJ/d, mean (SD))	6279 (1357)	7791 (1400)	9747 (2251)	<0.001	5142 (1171)	6420 (1162)	7851 (1780)	<0.001
Usual non-protein energy intake (kJ/d, mean (SD))	5283 (1290)	6454 (1378)	7893 (2045)	<0.001	4315 (1112)	5301 (1150)	6338 (1667)	<0.001
Usual protein intake (g/d, mean (SD))	59.7 (9.1)	80.1 (5.3)	111.1 (23.2)	<0.001	49.5 (7.8)	67.0 (4.2)	90.6 (15.6)	<0.001
Usual protein intake (g/kgBW/d, mean (SD))	0.7 (0.2)	1.0 (0.2)	1.3 (0.4)	<0.001	0.7 (0.2)	1.0 (0.2)	1.3 (0.4)	<0.001
Usual protein intake (% of energy, mean (SD))	16.3 (3.2)	17.7 (3.1)	19.5 (3.9)	<0.001	16.6 (3.2)	18.0 (3.2)	19.9 (4.2)	<0.001
Diet quality score (mean (SD))	65.8 (13.6)	68.4 (12.6)	68.4 (13.3)	<0.001	66.1 (12.7)	69.9 (11.9)	71.8 (12.4)	<0.001
Energy misreporting status (*n* (%))				<0.001				<0.001
Plausible reporters^d^	545 (44.4)	966 (78.7)	1022 (83.2)		565 (42.9)	1037 (78.7)	1070 (81.2)	
Misreporters	683 (55.6)	262 (21.3)	206 (16.8)		753 (57.1)	281 (21.3)	247 (18.8)	

BMI, body mass index; WC, waist circumference.

a Differences between tertiles for continuous variables were assessed by using analysis of variance. Differences between tertiles for categorical variables were assessed by using Pearson’s Chi-square test.

b Defined as BMI ≥ 25.

c Defined as WC ≥94 cm for males and ≥80 cm for females.

d Defined by using 1 SD cutoff for energy intake: energy expenditure between 53% and 147% for individuals with 1 recall day, and between 69% and 131% for individuals with 2 recall days.

### Association between protein intake and diet quality

Regression models resulted in statistically significant associations between protein intake expressed in three units and diet quality (P < 0.001), as shown in Table 2. Each g/d higher protein intake was associated with 0.15 (95% CI (0.12, 0.19)) unit higher DGI among males, and 0.25 (95% CI (0.22, 0.29)) unit higher DGI among females. Each per cent higher energy intake from protein was associated with 0.94 (95% CI (0.75, 1.13)) and 1.23 (95% CI (1.08, 1.38)) unit higher DGI among males and females, respectively. For each g/kgBW/d higher protein intake among males and females, there was a 7.49 (95% CI (5.19, 9.79)) and 9.67 (95% CI (8.00, 11.35)) unit higher DGI, respectively. In all models and units, the associations were stronger in females than males.

**Table 2.** Associations between protein intake and diet quality of Australian males and females^a^

	g/d			%EI			g/kgBW/d		
	Coeff.	95% CI	P value	Coeff.	95% CI	P value	Coeff.	95% CI	P value
Males									
Model 1	0.04	0.02, 0.06	**<0.001**	1.02	0.85, 1.19	**<0.001**	1.77	0.19, 3.35	**0.03**
Model 2	0.15	0.12, 0.18	**<0.001**	0.94	0.75, 1.13	**<0.001**	7.37	5.05, 9.70	**<0.001**
Model 3	0.15	0.12, 0.19	**<0.001**	0.94	0.75, 1.13	**<0.001**	7.49	5.19, 9.79	**<0.001**
Females									
Model 1	0.11	0.08, 0.14	**<0.001**	1.18	1.06, 1.30	**<0.001**	4.81	3.36, 6.25	**<0.001**
Model 2	0.25	0.22, 0.28	**<0.001**	1.23	1.07, 1.38	**<0.001**	9.57	7.89, 11.25	**<0.001**
Model 3	0.25	0.22, 0.29	**<0.001**	1.23	1.08, 1.38	**<0.001**	9.67	8.00, 11.35	**<0.001**

%EI, percent of energy intake.

a Diet quality was measured using the Dietary Guideline Index (range 0–130). Model 1 was adjusted for age, country of birth, socioeconomic status, and physical activity; Model 2 also included non-protein energy intake; and Model 3 also included non-protein energy intake and energy misreporting status.

Additional calculations using the average energy intake of Australian adult males in this analysis suggested a comparable increase in DGI using g/d and %EI units. Applying Model 3 to average 86-kg Australian adult males consuming 7939 kJ in a day, each gram higher protein intake is associated with a 0.15 DGI-units higher for males. Since a gram protein being equivalent to 17 kJ, a per cent higher daily energy intake from protein among males (79 kJ) equals to 4.67-g increase in protein, and this increase is correlated to 0.94 higher in DGI-unit. In other words, a g/d higher protein intake equates to 0.20 DGI-units higher among males.

### Association between protein intake and obesity

Linear regression models also demonstrated direct associations between protein intake and measures of obesity as shown in Table 3. For each g/d higher protein intake of males and females, BMI was higher by 0.07% (95% CI (0.04%, 0.11%)) and 0.09% (95% CI (0.04%, 0.15%)) kg/m^2^, respectively. Each per cent higher energy intake from protein was associated with 0.49% (95% CI (0.28%, 0.71%)) and 0.41% (95% CI (0.15%, 0.68%)) unit higher BMI among males and females, respectively. For each g/d higher protein intake, there was 0.04 cm (95% CI (0.01, 0.06)), and 0.05 cm (95% CI (0.00, 0.09)) higher in males and females WC, respectively. Each per cent higher energy intake from protein was associated with 0.26 cm (95% CI (0.11, 0.41)) higher WC among males only, but there was no association between protein intake expressed in %EI and WC among females. The sensitivity analysis adjusted for total energy intake instead of non-protein energy intake produced comparable coefficients, as provided in Supplementary Material 2.

**Table 3.** Associations between protein intake, BMI, and WC of Australian males and females^a^

	Males						Females					
	g/d			%EI			g/d			%EI		
	Coeff.	95% CI	P value	Coeff.	95% CI	P value	Coeff.	95% CI	P value	Coeff.	95% CI	P value
BMI^b^												
Model 1	–0.0000	–0.0003, 0.0003	0.92	0.0061	0.0040, 0.0083	**<0.001**	0.0001	–0.0004, 0.0005	0.82	0.0051	0.0025, 0.0077	**<0.001**
Model 2	0.0007	0.0003, 0.0010	**<0.001**	0.0050	0.0028, 0.0072	**<0.001**	0.0008	0.0003, 0.0013	**0.005**	0.0042	0.0016, 0.0068	**0.002**
Model 3	0.0007	0.0004, 0.0011	**<0.001**	0.0049	0.0028, 0.0071	**<0.001**	0.0009	0.0004, 0.0015	**0.001**	0.0041	0.0015, 0.0068	**0.003**
WC												
Model 1	–0.00	–0.03, 0.02	0.65	0.33	0.18, 0.48	**<0.001**	0.01	–0.02, 0.05	0.37	0.18	–0.01, 0.38	0.06
Model 2	0.03	0.01, 0.06	**0.01**	0.26	0.11, 0.42	**0.001**	0.04	–0.01, 0.08	0.09	0.19	–0.02, 0.40	0.07
Model 3	0.04	0.01, 0.06	**0.004**	0.26	0.11, 0.41	**0.001**	0.05	0.00, 0.09	**0.04**	0.19	–0.02, 0.39	0.08

BMI, body mass index (kg/m^2^); WC, waist circumference (cm); %EI, per cent of energy intake.

a Model 1 was adjusted for age, country of birth, socioeconomic status, and physical activity; Model 2 also included non-protein energy intake; and Model 3 also included non-protein energy intake and energy misreporting status.

b The interpretation of the β-coefficient estimates is 100 × (coefficient), referring to the percentage change for a 1-unit increase in protein intake with all other variables constant.

Multiple logistic regressions suggested direct associations between protein intake and overweight/obesity status as shown in Table 4. Odds ratios (ORs) of overweight/obesity status using BMI categories were comparable between g/d and %EI, and the associations varied across models and sexes. After additional adjustment for nonprotein energy intake and misreporting status, the OR of obesity was higher among those consuming more protein in g/d (males, OR = 1.01, 95% CI (1.00, 1.01); females, OR = 1.01, 95% CI (1.00, 1.01)). The OR was also higher among males and females consuming a larger per cent of energy from protein, but an additional adjustment to misreporting status produced a non-significant association in females.

**Table 4.** Associations between protein intake and obesity status of Australian males and females^a^

	Males						Females					
	g/d			%EI			g/d			%EI		
	OR	95% CI	P value	OR	95% CI	P value	OR	95% CI	P value	OR	95% CI	P value
Overweight/obesity												
Model 1	1.00	0.99, 1.00	0.52	1.06	1.03, 1.09	**<0.001**	1.00	1.00, 1.00	0.89	1.04	1.01, 1.06	**0.005**
Model 2	1.01	1.00, 1.01	**0.03**	1.04	1.01, 1.08	**0.02**	1.00	1.00, 1.01	0.08	1.03	1.00, 1.05	**0.047**
Model 3	1.01	1.00, 1.01	**0.03**	1.04	1.01, 1.07	**0.02**	1.01	1.00, 1.01	**0.04**	1.03	1.00, 1.05	0.056
Centrally overweight/obesity												
Model 1	1.00	0.99, 1.00	**0.04**	1.03	1.00, 1.06	**0.03**	1.00	1.00, 1.01	0.22	1.03	1.00, 1.05	**0.045**
Model 2	1.00	1.00, 1.00	0.73	1.02	0.99, 1.05	0.30	1.01	1.00, 1.01	0.06	1.03	1.00, 1.06	**0.04**
Model 3	1.00	1.00, 1.01	0.59	1.02	0.99, 1.05	0.30	1.01	1.00, 1.01	**0.03**	1.03	1.00, 1.06	**0.045**

%EI, percent of energy intake; OR, odds ratio; BMI, body mass index; WC, waist circumference.

a Overweight/obesity was defined as a BMI ≥ 25. Centrally overweight/obesity was defined as a WC ≥94 cm for males or ≥80 cm for females. Model 1 was adjusted for age, country of birth, socioeconomic status, and physical activity; Model 2 also included non-protein energy intake; and Model 3 also included non-protein energy intake and energy misreporting status.

Multiple logistic regressions also suggested direct associations between protein intake and central overweight/obesity status using WC categories, particularly in females. The OR of central overweight/obesity was higher in females consuming more g/d of protein (OR = 1.01, 95% CI (1.00, 1.01)), compared to females with less protein intake. The OR of central overweight/obesity was higher among females with a larger per cent of energy from protein (OR = 1.03, 95% CI (1.00, 1.06)), compared to those with a smaller proportion of energy from protein. Protein expressed in both units was positively associated with OR of central obesity in males, but the association was attenuated by adjustment for non-protein energy intake.

Additional calculations using the average energy intake of Australian males in this analysis showed a comparable increase in WC and BMI between protein units. For example, Model 3 showed that each g/d higher protein intake is associated with a 0.04-cm higher WC in males. Referring to 86-kg Australian males consuming 7939 kJ as an example, each per cent increase in energy from protein among males means that their 4.67-g increase in protein is correlated to 0.26-cm higher WC, which translates into approximately 0.06-cm higher WC per gram protein.

## Discussion

To our knowledge, this is one of the few studies assessing the associations of protein intake with diet quality and obesity using a nationally representative sample of Australian adults examining the impact of different ways of expressing protein intake. Protein expressed in g/d, g/kgBW/d, and per cent energy showed consistent direct associations with diet quality in males and females. Protein intake expressed in g/d or %EI also showed direct associations with obesity measures, yet the associations varied between sexes, which may be related to the diversity of protein food sources. While there appears to be a difference in effect sizes in protein associations with diet quality and obesity across measurement units, this is a result of the difference in unit scales. Compared to the absolute protein unit (g/d), relative measurement units (i.e. %EI and g/kgBW/d) require additional information and careful consideration when examining associations with diet quality and obesity.

### Association between protein intake and diet quality

Regression models in this study showed the direct association between total protein intake and DGI score among Australian males and females. This finding is in line with previous studies among Australians.^(12,13)^ A study using previous Australia’s National Nutrition Survey found a direct correlation between DGI score and protein intake (g/d) among women only, but a significant correlation was observed among both sexes after adjustment for energy intake.^(12)^ A cohort study among Australians aged ≥25 years also showed a direct correlation between total protein intake (%EI) and DGI and Mediterranean-Dietary Approaches to Stop Hypertension Diet Intervention for Neurological Delay Index (MIND) scores.^(13)^ However, no association was observed between protein intake and the Dietary Inflammatory Index score.^(13)^

Despite the consistent association between protein intake and diet quality, the effect size differed between sexes, and this difference might be due to the impact of plant and animal protein sources on overall dietary quality which may vary between sexes. For example, a previous study found that consuming higher protein diets was correlated with a higher total HEI score among young female American adults, but among males, these high-protein diets were only associated with higher HEI-component scores for dairy, total protein, and total vegetables.^(10)^ In this study, males generally scored higher in protein food components, such as meats, dairy, and alternatives, while females scored higher in fruit and vegetable components. However, some component scores varied within sex-specific tertiles of protein intake, such as males in the second tertile of protein intake scored highest for dairy component score and females in the second tertile scored highest for cereal/grain component score. This variation suggests that different amounts of certain protein food sources might contribute differently to diet quality scores, which warrants further investigation.

This study also found the different effect sizes across units, which are influenced by the scale of measurement units. Both g/d and %EI units produced similar effect sizes in both sexes when accounting for the unit scale, and this is not surprising given the adjustment for energy intake. However, it is important to note that all models in this study assume constant energy intake and contribution of other macronutrients, as done in the previous study.^(17)^ Therefore, the larger difference in effect sizes produced by g/d and %EI units is still possible with a higher intake of energy and other macronutrients.

Expressing protein intake in g/d will enable simple and direct interpretation of protein associations and diet quality, while relative units (i.e. g/kgBW/d and %EI) might require additional information on average energy intake and BW of study participants. Protein association with diet quality can still be captured using the g/kgBW/d unit, yet the results need to be interpreted cautiously as they might not represent the actual protein intake.^(18)^

### Association between protein intake and obesity

Both linear and logistic regressions in this study suggested the direct associations between protein intake and obesity measures, although there were no significant associations between protein with females WC when expressed in %EI. This was consistent with findings on %EI with the majority of previous studies,^(42,45–47)^ although other studies using the same unit found an inverse association^(48)^ or no association with measures of obesity.^(49)^ When protein consumption was expressed in g/d, the associations across most of the obesity measures were only significant after adjustment for energy intake and/or energy misreporting in the models. However, two previous studies reported no association between absolute protein intake (g/d) and obesity even after the adjustment for total energy intake.^(17,50)^

The varied protein associations with obesity between sexes might be related to the diverse protein food sources consumed by males and females that contribute differently to their energy intake. For example, the direct associations between animal protein intake and BMI in both sexes and WC among males reported in previous studies are somewhat explained by direct associations between animal protein and saturated fat intake, increased BMI, WC, and obesity risks.^(42,46,51)^ On the other hand, previous findings on inverse associations between plant protein intake with obesity measures in both sexes are related to the inverse associations between plant protein with energy, benefiting body composition and weight loss.^(42,51)^ Given that Australian males consumed high proportions of red and processed meats, while Australian females had high proportions of dairy, nuts, and seeds,^(52)^ total protein intake in this study might consist of different proportions of animal and plant protein sources between males and females and may therefore differentially influence associations with higher obesity measures. However, this warrants further investigation into how different food sources influence protein associations with obesity.

The different effect sizes between g/d and %EI units in protein association with obesity are also likely due to the scale of measurement units as those units produced similar effect sizes after accounting for the unit scale. This similarity is expected in isoenergetic models, where the models focus on investigating protein and remain agnostic to the changes of other macronutrients.^(18)^ Given the different influence of macronutrients on obesity or other health outcomes, statistical models are ideally adjusted for all macronutrients.^(44)^ However, the inclusion of macronutrients requires careful consideration, otherwise resulting in nonsensical models.^(18)^ The current study has shown that ‘all-components model’^(44)^ is not always possible due to potential multicollinearity among nutrients, and therefore adjustment for non-protein energy intake can be an option to accommodate other macronutrients in examining protein associations in different units with health outcomes.

### Strengths and limitations

Strengths of this study include the comparison of different units in influencing protein associations with diet quality and obesity. Another strength is the use of nationally representative data, followed by further estimation of usual dietary intake and stratification by sex. All models assessing diet quality and obesity were also adjusted for usual energy intake and energy misreporting status.

Diet quality in this study was measured using the DGI-2013, which performed well in studies examining diet quality relationships with BMI and health-related quality of life,^(25)^ and predicting risks of CVD and all-cause mortality among Australian adults^(26)^ DGI-2013 accounts for energy intake by considering different cutoffs of food intake between males and females rather than including the ratio of energy intake to energy expenditure in the scoring component, as done in the Australian Total Diet Score (TDS).^(53)^ Also, it is worth noting that many diet quality indices (e.g. DGI, TDS, and HEI) cap their highest scores but do not apply penalties for overeating, which therefore unable to show how far individuals exceed the recommended intake.^(8)^

Several limitations of this study should be considered. This analysis uses cross-sectional design, which does not allow any statements on causality. This analysis also used the survey data conducted more than 10 years ago, which therefore warrants further analyses when the new survey data are available. Another potential limitation of this study is the 18% missing anthropometric and dietary data which may have introduced sample bias. However, there were no differences between included and excluded individuals in terms of their sociodemographic characteristics (data not shown). It is also worth noting that while all analyses have been adjusted for age, this study did not explore the association across different age groups of adults. Given that older adults need higher protein intake for healthy ageing,^(22)^ further studies may consider age-stratification analyses in examining protein associations with health outcomes. Lastly, this study assumed low PAL due to the limited data available for physical activity measurement, which might lead to residual confounding. Therefore, future studies investigating protein intake may consider advanced methods for PAL measurement.

### Future directions/implications

Both absolute (g/d) and relative units (g/kgBW/d and %EI) can give similar results in protein associations with diet quality. However, the use of relative units needs additional information, such as average weight and energy intake, to prevent misleading interpretations of the associations. The use of g/kgBW/d unit in the population with overweight/obesity may also need further examination as the large denominator of the unit (i.e. high BW) may influence protein associations with diet quality.

Further research might specify models with different covariates and stratification in examining protein associations with health outcomes, such as stratifying analyses by age and including a more comprehensive measure of total daily physical activity. Statistical models will also ideally adjust for energy intake and other macronutrients.^(18)^ Adjustments for non-protein energy intake in this study were performed as the increased protein intake was the main interest while remaining agnostic of other macronutrients. This approach differs from previous studies that have adjusted for total energy intake to account for the joint effect between increasing protein and reducing other macronutrient intakes.^(43,50,54)^ However, we found adjustments for either non-protein energy intake or total energy intake produced similar results. Nonetheless, future research should consider how different approaches to energy adjustment may impact the interpretation of results.

The diverse food sources might explain the dissimilar associations in this study, which warrants further investigation. A high proportion of protein intake from animal sources is likely to contribute to high fat and energy intake and therefore increase BMI, WC, and the odds of obesity, while higher plant protein intake, depending on the specific food sources, might also come with lower fat and higher complex carbohydrates that help weight control.^(42,51)^ As protein associations with obesity and diet quality varied between sexes, future studies may also examine whether the consumption of different protein sources by males and females contributes to the varied associations.

## Conclusion

In conclusion, the effect size of the direct associations between total protein intake, diet quality, and obesity in males and females are influenced by measurement units. Three protein units consistently showed direct associations with diet quality, but the use of relative units in assessing protein associations with diet quality requires additional information. Both g/d and %EI also produce similar results when examining protein associations with obesity measures. The different associations across sexes are potentially due to diverse protein food sources, while the different effect sizes across protein units are influenced by the scale of measurement units. Adjustment for energy intake with careful consideration is also recommended when examining protein associations with diet quality and obesity. Future research may examine the influence of units on the associations between plant and animal protein with diet quality and obesity, as well as account for the different scales of those units.

## Supporting information

Arini et al. supplementary material 1 Arini et al. supplementary material

Arini et al. supplementary material 2 Arini et al. supplementary material

Arini et al. supplementary material 3 Arini et al. supplementary material

## Abbreviations

**%EI:** percent of energy intake; **ABS:** Australian Bureau of Statistics; **AMPM:** Automated Multiple 5-Pass Method; **ANOVA:** Analysis of variance; **BMI:** Body Mass Index; **CAPI:** Computer Assisted Personal Interview; **CATI:** Computer Assisted Telephone Interview; **CV:** Coefficient of variation; **CVD:** Cardiovascular disease; **DGI:** Dietary Guidelines Index; **g/d:** gram/day; **g/kgBW/d:** gram/kilogram body weight/day; **HEI:** Healthy Eating Index; **MIND:** Mediterranean-Dietary Approaches to Stop Hypertension Diet Intervention for Neurological Delay Index;**mTEE:** Measured total energy expenditure; **NNPAS:** National Nutrition and Physical Activity Survey; **OR:** Odds ratio; **PAL:** Physical activity level; **pTEE:** Predicted total energy expenditure; **rEI:** Reported energy intake; **SD:** Standard deviation; **SEIFA:** Socio-economic Indexes for Areas; **VIF:** Variance inflation factor; **WC:** Waist circumference.

## Supplementary material

The supplementary material for this article can be found at https://doi.org/10.1017/jns.2024.56
