# Supplementary material for: Association between protein intake, diet quality, and obesity in Australian adults: a comparison of measurement units
Source: J Nutr Sci. 2024 Sep 20;13:e42. doi: 10.1017/jns.2024.56 (PMC11428053; doi:10.1017/jns.2024.56)
Supplement: Arini et al. supplementary material 1 — Arini et al. supplementary material [file S2048679024000569sup001.docx]

Supplementary material 1. Components and scoring methods of Dietary Guideline Index

| **Dietary guideline** | **Food component and description** | **Criteria for minimum score** | **Criteria for maximum score*** | **Maximum score** |
| --- | --- | --- | --- | --- |
| 1. Enjoy a wide variety of nutritious foods | Food Variety**^†^** = Proportion of each food of 5 core food groups consumed at least 1 serve/week | 0% | 100% | 10 |
| 2. Plenty of vegetables | Vegetable intake = Total servings of vegetables consumed per day | 0 | 19-50 y: M ≥6, F ≥5  51-70 y: M ≥5.5, F ≥5  >70 y: M ≥5, F ≥5 | 10 |
| 3. Fruit | Fruit intake = Total servings of fruits consumed per day | 0 | ≥2 | 10 |
| 4. Grain (cereal) foods | a. Cereal/grain intake = Total servings of grains consumed per day | 0 | 19-50 y: M ≥6, F ≥6  51-70 y: M ≥6, F ≥4  >70 y: M ≥4.5, F ≥3 | 5 |
|  | b. High-fibre cereal intake = Type of bread usually consumed | White bread | Wholemeal bread | 5 |
| 5. Lean meat and poultry, fish, eggs, nuts and seeds, and legumes/beans | a. Total meat and alternatives intake = Total servings of lean meat and poultry, fish, eggs, nuts and seeds, and legumes/beans consumed per day | 0 | 19-50 y: M ≥3, F ≥2.5  51-70 y: M ≥2.5, F ≥2  >70 y: M ≥2.5, F ≥2 | 5 |
|  | b. Lean meat intake = Proportion of lean meats and alternatives to total meat and alternatives consumed per day | 0% | 100% | 5 |
| 6. Milk, yoghurt, cheese and/or their alternatives | Dairy and alternatives intake = Total servings of milk, yogurt, cheese and alternatives consumed per day | 0 | 19-50 y: M ≥2.5, F ≥2.5  51-70 y: M ≥2.5, F ≥4  >70 y: M ≥3.5, F ≥4 | 10 |
| 7. Drink plenty of water | a. Total beverages intake = Total servings of milk and soy beverages, smoothies, juices, low-calorie cordials and soft drinks, water, tea and coffee consumed per day | 0 | M ≥10, F ≥8 | 5 |
|  | b. Water intake = Proportion of water to total beverage intake per day | 0% | ≥50% | 5 |
| 8. Limit intake of foods containing saturated fat, added salt, added sugars  and alcohol | Total servings of discretionary foods intake = energy intake (kJ) from foods labelled as discretionary foods : 600kJ^‡^ | M >3, F >2.5 | M ≤3, F ≤2.5 | 10 |
| 9. Limit intake of foods high in saturated fat | a. Trimmed meat intake = Frequency of consuming lean meats and poultry containing <10% fat | Never or rarely | Usually | 5 |
|  | b. Reduced-fat milk intake = Type of milk usually consumed | Whole milk | Skim, low, or reduced-fat milk | 5 |
| 10. Small allowance of unsaturated oils, fats or spreads | Unsaturated spreads and oils intake = Total servings of unsaturated spreads and oils consumed per day | M >4, F >2 | 19-50 y: M ≤4, F ≤2  51-70 y: M ≤4, F ≤2  >70 y: M ≤2, F ≤2 | 10 |
| 11. Limit intake of foods and drinks containing added salt | a. Added salt during cooking based on salt use question whether salt added during cooking | Usually | Never or rarely | 5 |
|  | b. Added salt during meals based on salt use question whether salt added during meals | Usually | Never or rarely | 5 |
| 12. Limit intake of foods and drinks containing added sugars | Total servings of added sugar intake = grams of added sugar intake : 5g^§^ | M >1.5, F >1.25 | M ≤1.5, F ≤1.25 | 10 |
| 13. If you choose to drink alcohol, limit intake | Total servings of alcohol intake = grams of alcohol intake : 10g^\|\|^ | >2 | ≤2 | 10 |

y, years; M, male; F, female

*Criteria for maximum score were derived from the Australian Dietary Guidelines^(24)^ unless indicated otherwise

^†^Food variety score was based on the Recommended Food Score consisting of different varieties of fruits, vegetables, whole grains, lean meats and alternatives, low-fat dairy^(27)^

^‡^A serve of discretionary choices provides about 600 kJ^(28)^

^§^A serve of sugar contains about 5 grams of sugar^(20)^

^||^A serve of standard drink contains about 10 grams of alcohol^(28)^
