# Supplementary material for: Association between protein intake, diet quality, and obesity in Australian adults: a comparison of measurement units
Source: J Nutr Sci. 2024 Sep 20;13:e42. doi: 10.1017/jns.2024.56 (PMC11428053; doi:10.1017/jns.2024.56)
Supplement: Arini et al. supplementary material 2 — Arini et al. supplementary material [file S2048679024000569sup002.docx]

Supplementary material 2. Associations between protein intake, BMI, and WC of Australian males and females, adjusted for total energy intake*

|  | **Males** | | | | | | **Females** | | | | | |
| --- | --- | --- | --- | --- | --- | --- | --- | --- | --- | --- | --- | --- |
|  | **g/d** | | | **%EI** | | | **g/d** | | | **%EI** | | |
|  | **Coeff.** | **95% CI** | **p value** | **Coeff.** | **95% CI** | **p value** | **Coeff.** | **95% CI** | **p value** | **Coeff.** | **95% CI** | **p value** |
| **BMI**^†^ |  |  |  |  |  |  |  |  |  |  |  |  |
| Model 1 | **-**0.0000 | -0.0003, 0.0003 | 0.92 | 0.0061 | 0.0040, 0.0083 | **<0.001** | 0.0001 | -0.0004, 0.0005 | 0.82 | 0.0051 | 0.0025, 0.0077 | **<0.001** |
| Model 2 | 0.0007 | 0.0003, 0.0010 | **<0.001** | 0.0050 | 0.0028, 0.0072 | **<0.001** | 0.0008 | 0.0003, 0.0013 | **0.001** | 0.0042 | 0.0016. 0.0068 | **0.002** |
| Model 3 | 0.0007 | 0.0004, 0.0011 | **<0.001** | 0.0049 | 0.0028, 0.0071 | **<0.001** | 0.0009 | 0.0004, 0.0015 | **0.001** | 0.0041 | 0.0015, 0.0068 | **0.003** |
| **WC** |  |  |  |  |  |  |  |  |  |  |  |  |
| Model 1 | -0.01 | -0.03, 0.02 | 0.65 | 0.33 | 0.18, 0.48 | **<0.001** | 0.02 | -0.02, 0.05 | 0.37 | 0.18 | -0.01, 0.38 | 0.06 |
| Model 2 | 0.05 | 0.02, 0.07 | **0.002** | 0.29 | 0.14, 0.43 | **<0.001** | 0.04 | -0.00, 0.09 | 0.07 | 0.19 | -0.01, 0.39 | 0.06 |
| Model 3 | 0.05 | 0.02, 0.07 | **0.001** | 0.27 | 0.12, 0.41 | **<0.001** | 0.05 | -0.00, 0.10 | 0.06 | 0.16 | -0.04, 0.36 | 0.11 |

BMI, body mass index (kg/m^2^); WC, waist circumference (cm); %EI, percent of energy intake.

*****Model 1 was adjusted for age, country of birth, socioeconomic status, physical activity; Model 2 also included usual total energy intake; Model 3 also included usual total energy intake and energy misreporting status.

^†^The interpretation of the β-coefficient estimates is 100 x (coefficient), referring to the percentage change for a 1-unit increase in protein intake with all other variables constant.
