# Supplementary material for: Association between protein intake, diet quality, and obesity in Australian adults: a comparison of measurement units
Source: J Nutr Sci. 2024 Sep 20;13:e42. doi: 10.1017/jns.2024.56 (PMC11428053; doi:10.1017/jns.2024.56)
Supplement: Arini et al. supplementary material 3 — Arini et al. supplementary material [file S2048679024000569sup003.docx]

Supplementary material 3. DGI score of Australian males and females

| **Food components*** | **Male** | | | | **Female** | | | |
| --- | --- | --- | --- | --- | --- | --- | --- | --- |
|  | **T1** | **T2** | **T3** | **P value** | **T1** | **T2** | **T3** | **P value** |
| 1. Food Variety | 2.2 (1.0) | 2.6 (1.1) | 2.6 (1.1) | **<0.001** | 2.2 (1.0) | 2.7 (1.0) | 2.8 (1.0) | **<0.001** |
| 2. Vegetable intake | 6.1 (3.6) | 7.2 (3.2) | 7.1 (3.4) | **<0.001** | 6.2 (3.5) | 7.4 (3.1) | 7.6 (3.1) | **<0.001** |
| 3. Fruit intake | 5.9 (4.4) | 6.4 (4.3) | 6.1 (4.3) | **0.004** | 6.0 (4.1) | 6.8 (3.9) | 6.7 (3.9) | **<0.001** |
| 4a. Cereal/grain intake | 4.1 (1.4) | 4.4 (1.2) | 4.3 (1.3) | **<0.001** | 4.2 (1.3) | 4.5 (1.1) | 4.3 (1.3) | **<0.001** |
| 4b. High-fibre cereal intake | 2.2 (2.2) | 2.1 (2.1) | 2.2 (2.2) | 0.41 | 2.6 (2.2) | 2.5 (2.2) | 2.7 (2.2) | 0.28 |
| 5a. Meat and alternatives intake | 3.5 (1.8) | 4.4 (1.3) | 4.7 (1.0) | **<0.001** | 3.4 (1.8) | 4.3 (1.3) | 4.6 (1.0) | **<0.001** |
| 5b. Lean meat intake | 4.5 (1.2) | 4.4 (1.2) | 4.3 (1.2) | **0.041** | 4.5 (1.2) | 4.5 (1.1) | 4.5 (1.1) | 0.59 |
| 6. Dairy and alternatives intake | 5.9 (3.5) | 7.3 (3.3) | 7.1 (3.5) | **<0.001** | 4.1 (3.0) | 5.5 (3.2) | 6.0 (3.3) | **<0.001** |
| 7a. Total beverages intake | 4.4 (1.2) | 4.5 (1.0) | 4.4 (1.1) | **<0.001** | 4.6 (1.0) | 4.8 (0.8) | 4.7 (0.8) | **0.001** |
| 7b. Water intake | 4.9 (0.5) | 4.9 (0.5) | 4.9 (0.7) | 0.073 | 5.0 (0.4) | 5.0 (0.4) | 4.9 (0.4) | 0.67 |
| 8. Discretionary food intake | 1.2 (2.6) | 0.7 (2.1) | 0.9 (2.3) | **<0.001** | 1.5 (2.7) | 1.0 (2.4) | 1.3 (2.7) | **<0.001** |
| 9a. Trimmed meat intake | 2.4 (2.0) | 2.8 (1.8) | 3.1 (1.7) | **<0.001** | 2.2 (2.0) | 2.7 (1.8) | 3.0 (1.7) | **<0.001** |
| 9b. Reduced-fat milk intake | 1.6 (2.1) | 1.8 (2.1) | 1.6 (2.1) | **0.032** | 2.0 (2.2) | 2.3 (2.2) | 2.4 (2.2) | **<0.001** |
| 10. Unsaturated spreads and oils intake | 2.8 (3.5) | 1.9 (3.0) | 1.9 (3.0) | **<0.001** | 2.1 (3.2) | 1.2 (2.6) | 1.1 (2.6) | **<0.001** |
| 11a. Added salt during cooking | 3.4 (2.3) | 3.5 (2.3) | 3.5 (2.3) | 0.39 | 3.5 (2.3) | 3.5 (2.3) | 3.6 (2.2) | 0.27 |
| 11b. Added salt during meals | 4.2 (1.9) | 4.1 (1.9) | 4.1 (1.9) | 0.32 | 4.3 (1.7) | 4.3 (1.8) | 4.3 (1.7) | 0.75 |
| 12. Added sugar intake | 0.6 (2.0) | 0.4 (1.6) | 0.4 (1.6) | **0.007** | 0.5 (1.8) | 0.3 (1.4) | 0.4 (1.7) | **0.008** |
| 13. Alcohol intake | 6.0 (4.7) | 5.0 (4.8) | 5.4 (4.8) | **<0.001** | 7.3 (4.3) | 6.6 (4.5) | 6.9 (4.4) | **0.001** |

*Values are mean (SD). The within-sex comparison of mean scores between protein intake tertiles was assessed using analysis of variance
